# Response of a Zn_2_TiO_4_ Gas Sensor to Propanol at Room Temperature

**DOI:** 10.3390/s17091995

**Published:** 2017-08-31

**Authors:** Ibrahim Gaidan, Dermot Brabazon, Inam Ul Ahad

**Affiliations:** 1 Faculty of Engineering, Electrical & Electronics Eng. Department, Sirte University, Sirte, Libya; 2 Advanced Processing Technology Research Centre, Dublin City University, Dublin 9, Ireland; dermot.brabazon@dcu.ie (D.B.); inamul.ahad@dcu.ie (I.U.A.)

**Keywords:** gas sensors, XRD, ZnO and TiO_2_

## Abstract

In this study, three different compositions of ZnO and TiO_2_ powders were cold compressed and then heated at 1250 °C for five hours. The samples were ground to powder form. The powders were mixed with 5 wt % of polyvinyl butyral (PVB) as binder and 1.5 wt % carbon black and ethylene-glyco-lmono-butyl-ether as a solvent to form screen-printed pastes. The prepared pastes were screen printed on the top of alumina substrates containing arrays of three copper electrodes. The three fabricated sensors were tested to detect propanol at room temperature at two different concentration ranges. The first concentration range was from 500 to 3000 ppm while the second concentration range was from 2500 to 5000 ppm, with testing taking place in steps of 500 ppm. The response of the sensors was found to increase monotonically in response to the increment in the propanol concentration. The surface morphology and chemical composition of the prepared samples were characterized by Scanning Electron Microscopy (SEM) and X-Ray Diffraction (XRD). The sensors displayed good sensitivity to propanol vapors at room temperature. Operation under room-temperature conditions make these sensors novel, as other metal oxide sensors operate only at high temperature.

## 1. Introduction

The detection and quantification of alcohols with high sensitivity and selectivity is required in various industrial sectors, such as the pharmaceutical, chemical, clinical laboratories, agro-food and alcohol-based fuel industries. A number of conventional spectroscopic techniques are currently used in industries for accurate alcohol detection. However, these techniques often require complex sample preparation steps, and therefore need highly skilled and trained operators, and are not suitable for rapid onsite measurements. Due to complexity of the instruments and the high costs, the requirement for highly sensitive and selective alcohol sensors with real-time monitoring capabilities has increased. Alcohol sensors can be categorized on the basis of the type of transducer used to convert the alcohol content information into an electrical signal. Chemical- and enzyme-based biosensors are two major classes of alcohol sensors. Biosensors are highly selective in nature, due to the specific reaction between the enzyme and the tested alcohol; they are not suitable for long-term use, and have limited shelf-life. Chemical sensors demonstrate high sensitivity, but are prone to poor selectivity. Semiconducting metal oxide composite materials have been used as the sensing element for fabrication of thin film chemical alcohol sensors. Thin film metal oxide sensors are inexpensive, easy to fabricate, demonstrate long term stability, and provide real-time monitoring. However, metal oxide sensors require high operating temperatures as the sensing material needs to be heated up to several hundred degrees to transform from insulator to semiconductor. For example, the sensitivity of SnO_2_- and In_2_O_3_-based sensors with platinum interdigitated electrodes on alumina substrates for detecting ethanol was investigated over a range of 10–5000 ppm [1]. A TiO_2_ thin film was used to detect alcohol by Sberveglieri et al. [2]. This sensor was fabricated on alumina substrate with Pt interdigitated electrodes using a sol-gel technique. The sensor was used to detect methanol, ethanol and propanol at 2100, 2000 and 2600 ppm at operating temperatures of 300, 400 and 500 °C, respectively. In another study, SrFeO_3_ oxide P-type semiconductor gas sensor was investigated. A solid-state reaction method was used to deposit the sensing material. The sensor worked at operating temperatures of 275 to 375 °C and a concentration range of 0–2000 ppm [3]. This sensor demonstrated good sensitivity and selectivity to the vapors of Ethanol (C_2_H_5_OH). The effect of adding CdO to ZnFe_2_O_4_-based semiconductors for improved sensitivity in air and for ethanol was reported [4]. A Zn_2_TiO_4_ ethanol gas sensor was investigated. The sensor was used at concentration ranges of 50 and 200 ppm at 300–500 °C [5]. TiO_2_ thin film-based gas sensors were demonstrated for detection of methanol, ethanol and benzene at room temperature [6]. Titanium and tungsten oxide-based gas sensors were used to detect NO_2_ and CO [7]. Nb-doped titanium dioxide thin films with a thickness of 300 nm, deposited on Al_2_O_3_ substrate, were used to detect nitrogen dioxide (NO_2_) [8]. The sensitivity of the sensor to NO_2_ was recorded to be 1.0/ppm at 350 °C. Improved sensitivity of ZnO nano-wires on hydrogen sulfide (H_2_S) was also demonstrated, emphasizing the effect of nano-size effect on producing high response in presence of even trace-level of a gas [9]. The sensor was used to detect different gases, and showed good sensitivity and selectivity to H_2_S at low concentrations (5 ppb). The sensor was tested at an operating temperature of 300 °C. A micro hotplate was used to control the temperature. TiO_2_ thin film for detecting NO_2_ was investigated [10]. A ZnO sensor was used to detect O_3_ (0.1 ppm) [11]. The sensor exhibited the highest sensitivity to O_3_ at 250 °C. A gas sensor based on Zn_2_TiO_4_ as a sensing material for ethanol was studied [5]. The sensor was tested at operating temperatures of 300–500 °C, and exhibited the highest sensitivity at 500 °C. TiO_2_ and ZnO, or mixture of both, have been used by many researchers to detect different gases. For example, TiO_2_/ZnO has been used to detect ethanol [12,13], ZnO to detect NO_2_ [14], and TiO_2_ to detect chloroform [15].

Some mixture formulas, such as xCO_2_O_4_, xFe_2_O_4_ and xTiO_4_ (x = Ni, Cu, Zn, Zn_2_, Sn, Cd, Mg, etc.), have become more common to use in gas sensing applications. xCo_2_O_4_ (x = Ni, Cu, or Zn) was used to detect CH_3_COOH at 300 °C [16]. ZnCo_2_O_4_ and Zn_2_SnO_4_/SnO_2_ gas sensors were investigated to detect CO, C_3_H_8_ and NO_2_ at 600 °C [17]. A ZnCo_2_O_4_ sensor exhibited a good sensitivity to LPG at both high and low temperatures. The sensor was tested at (room temperature −400 °C). The highest sensitivity to 40 ppm LPG was recorded at 250 °C [18]. A NiCo_2_O_4_ sensor was studied for detecting O_3_ [19]; the sensor demonstrated the highest sensitivity at 200 °C. Table 1 presents a brief list of selective research studies on different metal oxide composite sensing materials developed to test selected gases.

It can be concluded from the research studies that a high operating temperature is required for transforming the metal oxides from insulator to semiconductor state. Hotplates are normally used to increase the temperature of the sensing material for sensing the desired gas. This results in high power consumption, oxidation of sensing materials, and limited use of the sensing materials for miniaturized sensing platforms and wearable sensors. Additionally, gold or platinum electrodes are commonly used, which makes these sensors expensive.

These limitations of metal oxide-based sensors have encouraged researchers to use polymer gas sensors, which work at room temperature with low power consumption. For example, to detect methanol, ethanol and benzene at room temperature, a TiO_2_ dispersed in poly vinylidenflouoride (PVDF) gas sensor was used [6]. The film was deposited on glass substrate with gold electrodes. The sensor not only demonstrated a great sensitivity to methanol, ethanol and benzene gases at low concentrations, but longer response (2 min) and recovery (6 min) times were also recorded. Although the shelf-life of conductive polymer-based metal oxide gas sensors is limited compared to the above-mentioned sensors, they still have a longer lifetime than enzyme-based biosensors [20]. In previous studies by our group, NiFe_2_O_4_ and ZnFe_2_O_4_ gas sensors were developed and tested for sensing methanol, ethanol, and propanol at room temperature [21,22,23].

In the current study, new composite materials were developed by mixing different weight percentages of TiO_2_ and ZnO to fabricate a novel gas sensor with high sensitivity towards alcohols. The conductive polymer polyvinyl butyral (PVB) was mixed with the metal oxide composites, along with carbon black, to increase the porosity and make them operational at room temperature. The morphological and chemical characterizations of the prepared samples were examined, and the developed sensors were tested for sensing propanol vapors using a specially designed gas test chamber. The response of the developed sensors was recorded for propanol vapors at two concentration ranges (500 to 3000 ppm and 2500 to 5000 ppm) at room temperature.

## 2. Experimental Work

**Figure 1:**
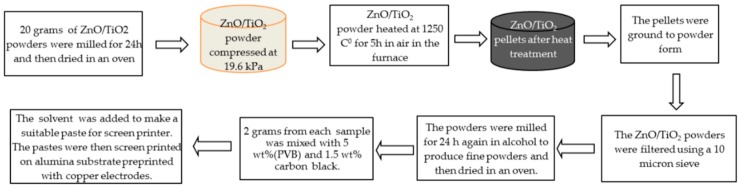
Schematic indicating the flow of the implemented experimental procedure steps.

Titanium dioxide (TiO_2_), zinc oxide (ZnO) and carbon black was supplied by Alfa Aesar. Figure 1 shows the experimental working steps. Three powder samples with different molecular weight percentage of TiO_2_ and ZnO were prepared according to Table 2. Samples were prepared using 75, 50 and 25 Mwt % of TiO_2_, mixed with 25, 50 and 75 Mwt % of ZnO. The powders were mixed with wet-ball milled in alcohol for 24 h, dried in an oven at 120 °C, and then compressed at 2 ton to form pallets. The pallets were heated in a furnace at 1250 °C at a rate of 5 °C/min, followed by a cooling rate of 3 °C/min in air. The solid pallets were then broken up and ground down to powder form using mortar and pestle. The powders were filtered with a 10 micron sieve. The powders were mixed again with a wet-ball mill in alcohol (isopropanol) and then dried again in an oven to produce fine powders.

**Figure 2:**
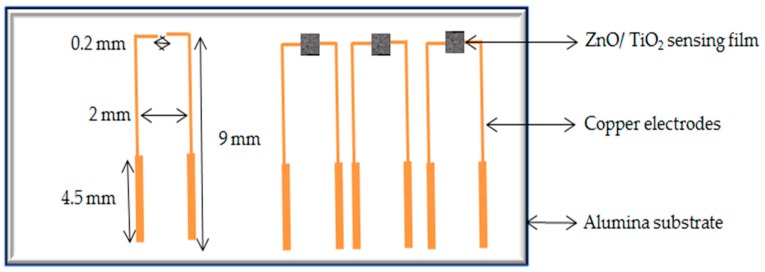
Configuration of the ZnO/ TiO_2_ sensors.

Two grams of prepared powder from each sample was mixed with 5 wt % of polyvinyl butyral (PVB) and 1.5 wt % carbon black. PVB was used as a binder and carbon black was used to increase the conductivity of the films. Ethylen-glycol-monobutyl-ether was used as a solvent for PVB to make pastes for screen printing. To fabricate sensors, alumina substrates were used to deposit copper electrodes using a novel approach as described previously [48]. The prepared pastes were then screen printed on the top of thin copper electrodes on alumina substrate using a DEK RS 1202 automatic screen printer. Figure 2 presents the configuration of the developed sensors. SEM images were obtained for the mixed powders and screen-printed thin films. XRD measurements (from 10° to 70°, 2θ) were also performed on the screen-printed sensing materials to examine the final compositions.

A gas testing chamber, designed and developed previously, was used to test the response of the three screen-printed sensors with different molecular weights of metal oxides. This specially designed gas testing chamber enable measurement of different gases with predefined concentrations using screen-printed sensors.

## 3. Results and Discussion

### 3.1. SEM and XRD Results

**Figure 3:**
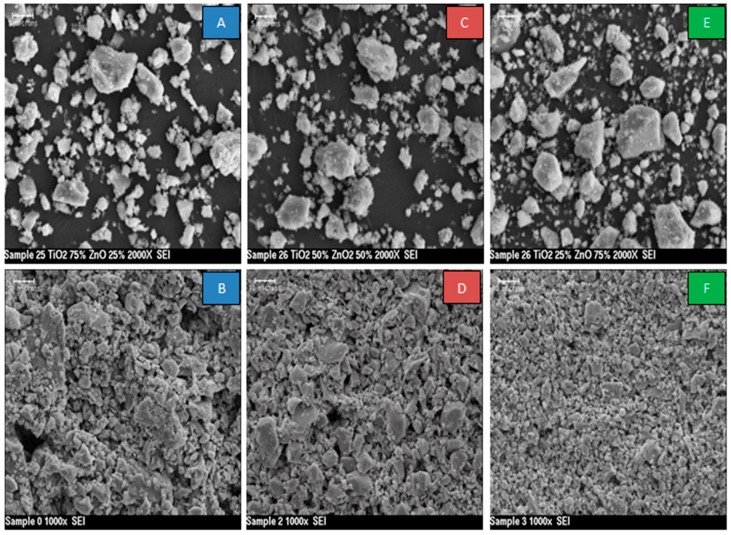
SEM images of TiO_2_/ZnO mixed powder (**A**,**C**,**E**) and screen-printed sensing material (**B**,**D**,**F**) for Sample 1–3 respectively.

Figure 3A,C,E shows the SEM images for the powders of sample 1, 75/25; sample 2, 50/50; and sample 3, 25/75 TiO_2_/ZnO Mwt% and Figure 3B,D,F shows the corresponding screen-printed thin films. The SEM images of powders from all three samples demonstrated similar morphology. The powder grain size was typically between 2 and 5 microns. The SEM images of the screen-printed films (Figure 3B,D,E) demonstrated uniform deposition of the sensing material. The samples displayed a granular structure and good porosity. For Sample 1, with the highest TiO_2_ contents, a large granular and porous structure was observed. For Sample 2, the screen-printed film showed a similar structure to sample one. The grain size was smaller than that of the Sample 1 sensor; however, the porosity was similar. In the third sample, a fine granulated structure was observed with an average grain size of 1 micron to 5 microns. 

**Figure 4:**
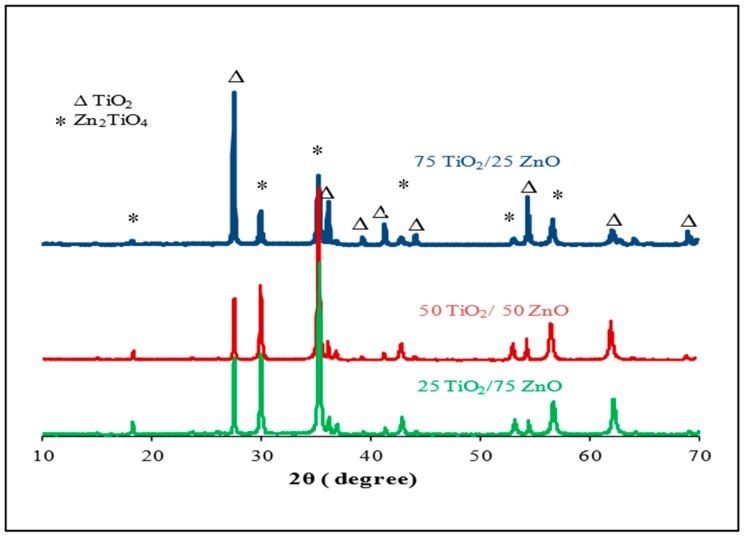
The XRD patterns from 10° to 70°, 2θ, for TiO_2_/ZnO samples, after being heated at 1250 °C for 5 h.

Figure 4 shows the XRD patterns of screen-printed samples from 10° to 70°, 2θ. The results were analyzed using JCPDs files No.: 18-1487 and 01-1292 for Zn_2_TiO_4_ and TiO_2_, respectively. XRD data of the samples showed that, after heating and screen printing, the ratio of Zn_2_TiO_4_ to TiO_2_ depended mainly on the ratio of TiO_2_ to ZnO used in the preparation of the powder mixtures before heating. XRD data for Sample 2 and Sample 3, with 50/50 and 25/75 Mwt % of TiO_2_/ZnO, showed that the highest intensity was observed for the peak associated with Zn_2_TiO_4_. However, in the case of Sample 1 (with 75/25 Mwt % TiO_2_/ZnO), the highest intensity in the final composition was observed for TiO_2_. The differences observed in the final compositions were due to solid-state reactions between both oxides during heating, and the ratio of oxides in the mixtures. Therefore, many characteristic peaks of TiO_2_ were observed in the final composition of Sample 1. The XRD results of Zn_2_TiO_4_ were in good agreement with the previous studies [5,49,50,51].

### 3.2. Response of the Sensors to Propanol

**Figure 5:**
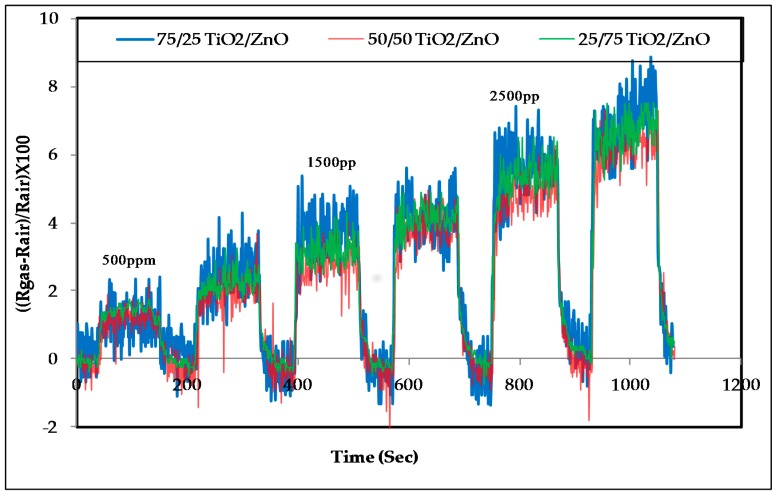
The response of the Zn_2_TiO_4_ sensors to propanol at concentration range 500–3000 ppm increasing with a step size of 500 ppm at room temperature.

**Figure 6:**
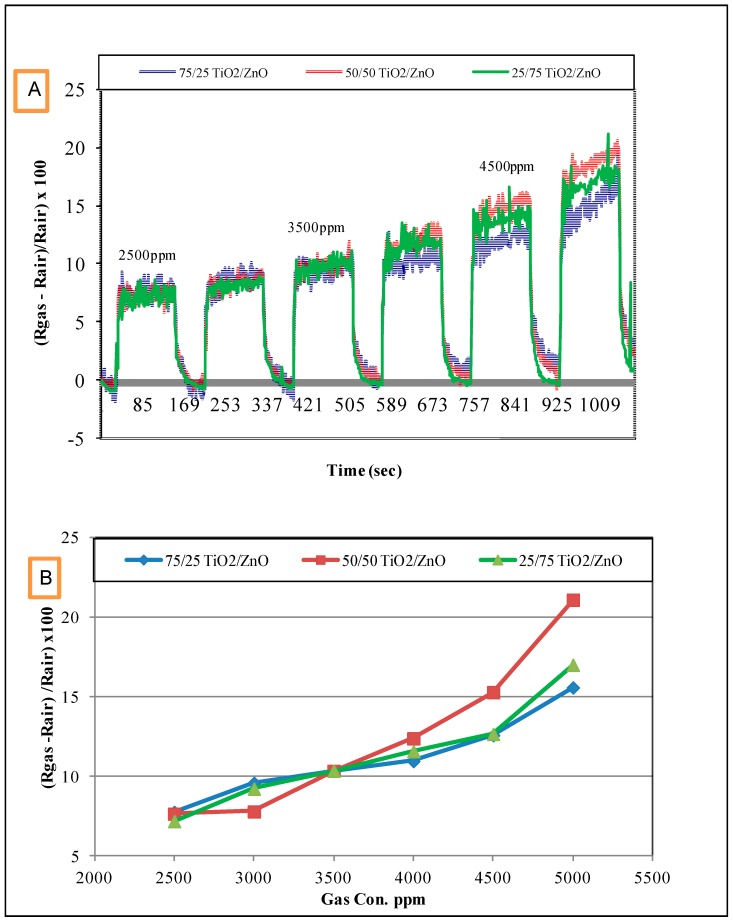
The response of the Zn_2_TiO_4_ sensors to propanol at a concentration range of 2500–5000 ppm at room temperature. (**A**) The response of the sensors with time (Sec.) and (**B**) The response of the sensors with gas concentration (ppm).

Figure 5 shows the response of the sensors to propanol vapors at room temperature in increasing steps of 500 ppm at a concentration range of 500–3000 ppm. Figure 6A,B illustrates the response of the sensors to propanol vapors at room temperature at a concentration range of 2500–5000 ppm. The response for the sensors was calculated using the following relationship:

(1) $$\Delta R=\left(\frac{R_{gas}-R_{air}}{R_{air}}\right)\times 100$$

where Δ*R* is change in resistance, *R_gas_* is sensor resistance when exposed to gas, and *R_air_* is base resistance of the sensor in air.

From these results, it can be concluded that:

- The response of the sensors increased as the gas concentration increased;
- However, no significant difference was observed in the response of the three sensors at low concentrations; sensor 2 (50/50 TiO_2_/ZnO) had the highest sensitivity for propanol, followed by sensor 3 and then sensor 1 at higher concentrations (above 4000 ppm);
- This difference in sensor response could be associated with the final composition of the sensing materials. The XRD results proved that the peaks of Zn_2_TiO_4_ were higher for Samples 2 and 3 (the highest density) than for Sample 1. The TiO_2_ was at the highest density in Sample 1. In addition to this, the surface morphology of the three samples was also different in terms of the number, size and shape of the pores on the surface of the films (see Figure 3);
- The signal noise was high in the lower concentration range (500–2500 ppm) compared with the response of the same sensors for the higher concentration range (2500–5000 ppm). This high noise could be attributed to the effect of a slight change in the working temperature or the effect of some other gases that were already in the air of the gas testing chamber.

In a previous study, Zn_2_TiO_4_ particles were prepared using the ball-milling technique, and deposited as a thick film to detect alcohol gases (ethanol) at 50 and 200 ppm over an operating temperature range of 300–500 °C [5]. The highest sensitivity of the sensor was observed at 500 °C. The sensor was developed without using any polymer binder, and can only be used for high temperature applications. ZnFe_2_O_4_ and NiFe_2_O_4_ thick films were also used in previous studies by our group to detect methanol, ethanol and propanol at room temperature [21,22,23,52]. In the current study, Zn_2_TiO_4_ exhibited good sensitivity to propanol at room temperature. The response of the sensor depends on the final composition of the sensing material. In this study, no significant effect was observed in the final composition of the response, particularly at lower concentrations, as can be seen in Figure 5 and Figure 6.

It is well known that the interaction of a gas on the sensing material surface depends on the type of the sensing material (p- or n-type) and type of testing gas (oxidation or reduction gases). These two factors provide the basis for the increment or decrement in the resistivity of films. 

The reaction of the gas on the surface of metal oxides has been reported in many studies [5,11,13,15,24]. However, the mechanism of the reaction of the gases on the surface of the films is still not well understood. In particular, for the sensors developed and tested in the current study, the interaction mechanism and conductivity response would be influenced by the complicated chemistry of the sensing material, which consists of composite metal oxides, polymer, and carbon black. Further detailed studies will be performed to investigate the adsorption of gas molecules on to the sensing material surface. 

The Zn_2_TiO_4_ sensors developed in this study were novel in terms of composition. Conductive polymer was used as binder to combine the oxides and adhere it to the substrate. This study indicates that the use of the conductive polymer increased the pores in the surface of the films, which increased the sensitivity of the sensors at room temperature without heating the sensing material. Therefore, these sensors can be used for real-time monitoring of propanol with lower power consumption requirements; thus, they can be used in miniaturized sensing platforms and wearable sensing systems. Additionally, the use of copper electrodes makes the sensor more cost effective and able to interface with electronic circuits in simple configurations.

## 4. Conclusions

In the current work, the composition of ZnO/TiO_2_ was varied significantly from those reported in the literature and, as a result, properties were observed that were different from those previously reported. Thick film gas sensors, based on a Zn_2_TiO_4_ semiconductor with different ZnO and Ti_2_O contents were developed and tested for sensing propanol vapors. The electrochemical results from these sensors showed:

- They had good sensitivity to propanol vapor at room temperature;
- The response of the sensors monotonically increased as the gas concentration increased;
- XRD measurements of the screen-printed sensing materials illustrated that the final compositions (Zn_2_TiO_4_ to TiO_2_) depended on the ratio of TiO_2_ to ZnO used in preparing the mixtures before being heated;
- SEM images of the films showed granular microstructure ranging from 2–5 microns;
- Using copper as an electrode material makes sensors cost effective compared to sensors with Pt and Au electrodes;
- The developed sensors consume very low power compared with those sensors reported in the literature in [5,12], as heating of the sensing material is not required;
- The simple structure of the sensor with copper electrodes makes it easy to interface with electrical systems;
- The developed sensors can be used in various applications at room temperature, including as gas safety monitors;
- Further detailed studies are required to better understand the interaction mechanism between the sensing material and the tested gas.

## Figures and Tables

**Table 1:** List of selected sensing materials for sensing different gases.

Sensing Materials	Sensitive Gases	Year/Reference
ZnO	H_2_S	2017 [9]
ZnO–SnO_2_	O_3_	2016 [24]
TiO_2_	NO_2_	2015 [10]
Zn_2_TiO_4_	C_2_H_5_OH	2015 [5]
ZnO	NO_2_	2013 [14]
Zn/Zn_2_SnO_4_ + SnO_4_	CO/C_3_H_8_	2013[17]
TiO_2_	Chloroform	2014 [15]
TiO_2_ and ZnO	C_2_H_5_OH	2012 [13]
TiO_2_-ZnO	NO2	2010 [12]
ZnO	C_2_H_5_OH	2010 [25]
SnO_2_ and Pt/SnO_2_	CO	2006 [26]
ZnO	C_2_H_5_OH and Acetone	2006 [27]
SnO_2_	H_2_S and CO	2006 [28]
SnO_2_	O_3_, COand NO_2_	2006 [29]
SnO_2_	O_3_ and NO_2_	2006 [30]
SnO_2_/Al/Ni	LPG gas	2006 [31]
SnO_2_ or Ga_2_O_3_	CO and CO_2_	2005 [32]
SnO_2_	H_2_, C_2_H_5_OH CH_4_	2005 [33]
SnO_2_	CO and CO_2_	2005 [34]
WO_3_	Hydrocarbon gases	2005 [35]
TiO_2_	CO	2004 [36]
Fe_2_O_3_–SnO_2_	NO_2_ and C_2_H_5_OH	2004 [37]
Pt-SnO_2_	C_2_H_5_OH	2004 [38]
SnO_2_	C_4_H_10_	2004 [39]
SnO_2_–ZnO and SnO_2_–ZnO–CuO	H_2_S LPG, NO*_x_*, CO_2_, CO and CH_4_	2004 [40]
SnO_2_–Co_3_O_4_	CO and H_2_	2004 [41]
CdSnO_3_	Cl_2_	2004 [42]
BaSnO_3_	H_2_S, CH_3_SH	2004 [43]
ZnO–TiO_2_	Alcohol, Acetone, Benzene, Toluene and xylene	2004 [44]
MFe_2_O_4_	H_2_S, CH_3_SH	2003 [45]
NiO-doped WO_3_	NO_2_	2003 [46]
WO_3_	H_2_S	2001 [47]

**Table 2:** Description of powder samples prepared for developing screen printing paste.

Sample	TiO_2_ (Mwt %)	ZnO (Mwt %)
Sensor 1	75	25
Sensor 2	50	50
Sensor 3	25	75
